# Disrupting CCT-*β *: *β*-tubulin selectively kills CCT-*β* overexpressed cancer cells through MAPKs activation

**DOI:** 10.1038/cddis.2017.425

**Published:** 2017-09-14

**Authors:** Yan-Jin Liu, Vathan Kumar, Yuan-Feng Lin, Po-Huang Liang

**Affiliations:** 1 Institute of Biological Chemistry, Academia Sinica, Taipei 11529, Taiwan, ROC; 2 Graduate Institute of Clinical Medicine, College of Medicine, Taipei Medical University, Taipei 11031, Taiwan, ROC; 3 Institute of Biochemical Sciences, National Taiwan University, Taipei 10617, Taiwan, ROC

## Abstract

We have previously demonstrated the ability of I-Trp to disrupt the protein–protein interaction of *β*-tubulin with chaperonin-containing TCP-1*β* (CCT-*β*). This caused more severe apoptosis in multidrug-resistant MES-SA/Dx5, compared to MES-SA, due to its higher CCT-*β* overexpression. In this study, we screened a panel of cancer cell lines, finding CCT-*β* overexpression in the triple-negative breast cancer cell line MDA-MB-231, colorectal cancer cell lines Colo205 and HCT116, and a gastric cancer cell line MKN-45. Thus, I-Trp killed these cancers with sub- to low-*μ*M EC_50_, whereas it was non-toxic to MCF-10A. We then synthesized analogs of I-Trp and evaluated their cytotoxicity. Furthermore, apoptotic mechanism investigations revealed the activation of both protein ubiquitination/degradation and ER-associated protein degradation pathways. These pathways proceeded through activation of MAPKs at the onset of CCT-*β *: *β*-tubulin complex disruption. We thus establish an effective strategy to treat CCT-*β* overexpressed cancers by disrupting the CCT-*β *: *β*-tubulin complex.

Molecular chaperones play a central role in maintaining protein homeostasis through an intricate system of cooperative mechanisms that balance protein biosynthesis, folding, translocation, assembly/disassembly, and clearance. They can be classified into two mechanistic classes: (1) chaperones that promote folding of non-native proteins by binding to and releasing their substrates into the bulk matrix of cells, and (2) chaperones that promote folding by sequestering single protein molecules within a molecular cage (chaperonins).^1^ The former includes most heat shock proteins (HSPs), such as HSP70 and HSP90, whose expression is upregulated in response to environmental stress. The latter, the eukaryotic chaperonin family, includes the type I chaperonin, HSP60, and the type II hetero-oligomeric chaperonin, TRiC (T-complex protein-1 ring complex), also known as chaperonin-containing TCP-1 (CCT). CCT is a chaperone that assists in the folding of the cytoskeleton proteins, actin and tubulin,^2^ and intracellular proteins such as cyclin E1,^3^ histone deacetylase,^4^ and protein phosphatase PP2A regulatory subunit B.^5^ CCT contains eight different subunits (*α*, *β*, *γ*, *δ*, *ε*, *ζ*, *η*, and *θ* or 1–8), each with an approximate molecular weight of 60 kD. These eight subunits have a fixed order, arranged in two back-to-back rings, and are stacked conversely to form an enclosed cavity where protein folding takes place.^6, 7^ Different subunits share only ~30% identity in amino-acid sequences,^8^ and the difference increases its substrate variety and specificity. Emerging evidence implicates CCT in the pathogenesis of numerous cancers. Importantly, several proteins associated with tumor-genesis have been identified as CCT clients, including signal transducer and activator transcription 3 (STAT3), cyclins B and E, P53 and Von Hippel-Lindau.^3, 9, 10, 11^

CCT’s interaction with tubulin has been illustrated using cryoelectron microscopic (CryoEM) analysis.^12^ This docking model reveals that the CCT-*β* subunit binds with the fragment V353-P357 within *β*-tubulin’s C-terminal region to stabilize microtubular structure. Drug-resistant MES-SA/Dx5 cells have a higher endogenous CCT-*β* level and are therefore more vulnerable than MES-SA cells to a peptide disrupting the protein–protein interaction (PPI).^13^ A compound, I-Trp with iodomethyl ketone warhead (denoted as **1a** in Figure 1), was found to alkylate Cys^354^ of *β*-tubulin, disrupt the PPI, and induce cell apoptosis.^13^ The anti-cancer drug Taxol targets *β*-tubulin at the ATP-binding site. In contrast, I-Trp targets *β*-tubulin in the PPI interface between *β*-tubulin and CCT-*β*. I-Trp treatment activates both the Hsp90-associated protein ubiquitination/degradation pathways, which eliminate misfolded proteins in the cytoplasm, and the valosin-containing protein-centered endoplasmic reticulum-associated protein degradation (ERAD) pathway, which reduces the excessive levels of unfolded polypeptides from ER. Activating these degradation pathways leads to caspase activation and cell apoptosis.^14^ However, the signaling pathways involving pro- and anti-apoptotic proteins as well as the kinases have not yet been investigated.

In this study, we identified four cancer cell lines overexpressing CCT-*β* and determined the effect of I-Trp in these cancer cell lines. Immunoprecipitation assay in these cells confirmed the disruption of the CCT-*β *: *β*-tubulin complex by I-Trp. Our mechanistic studies show that I-Trp led the cancer cells toward ER stress with elevated intracellular Ca^2+^ levels and mitochondrial destruction, ultimately activating caspases for apoptosis. During ER stress, the kinase functions of PKR-like ER kinase (PERK) and inositol requiring enzyme 1 (IRE1)-*α* involving the MAPK family, such as ERK, p38, and JNK, were activated. This study thus establishes CCT-*β*: *β*-tubulin as a novel drug target for combating malignant cancers overexpressing CCT-*β*.

## Results

### I-Trp-induced apoptosis in the selected cancer cell lines

We screened a variety of different cancer cell lines and found that a number of them express CCT-*β* at higher levels, including triple-negative breast cancer (TNBC) MDA-MB-231, colorectal cancers HCT116 and Colo205, and gastric cancer MKN-45 (Figure 2a). Western blotting revealed that these cancer cell lines had much higher CCT-*β* expression levels than the non-tumorigenic epithelial MCF-10A (Figure 2a).

To evaluate the potency of I-Trp in killing these cancer cell lines, we performed MTT assay to determine the cell viability of target cells. Treating these cells with I-Trp for 72 h significantly decreased the cell viability in a dose-dependent manner (0.31–20 *μ*M). The EC_50_ values of I-Trp against HCT116, Colo205, MKN-45, and MDA-MB-231 cells were 0.88, 0.90, 1.27, and 2.50 *μ*M, respectively, whereas the EC_50_ for MCF-10A was higher than 20 *μ*M (Figure 2b). The cancer cells with higher expression levels of CCT-*β* were indeed more sensitive to I-Trp.

Furthermore, to confirm the correlation of I-Trp cytotoxicity and the levels of CCT-*β*, we performed CCT-*β* knockdown experiments using two independent CCT-*β* shRNA clones and then determined the I-Trp-induced toxicity in MDA-MB-231 breast cancer cells. As shown in Figure 2c, the knockdown of CCT-*β* (top panel) significantly (*P*<0.001) reduced cell death from I-Trp exposure.

In order to ascertain whether I-Trp-induced cytotoxicity through an apoptotic mechanism, we analyzed the population of sub-G0 hypodiploid cells by flow cytometry. I-Trp caused dose-dependent sub-G0 cell accumulation of these cancer cells at the indicated concentration ranges (Figure 2d). We then stained for active caspase-3 to determine the apoptotic cells by flow cytometry. As shown in Figure 2e, after 72 h of treatment with different EC_50_ concentrations of I-Trp on the four cancer cell lines, the fluorescence signals (shifted to right) corresponding to the active caspase-3 were increased, indicating induction of apoptosis.

Next, we investigated the long-term effect of I-Trp on proliferation of the target cells using the colony formation assay. After these cancer cells were seeded at low density in normal serum medium for 7 days, the I-Trp-treated cells formed more diffuse or smaller colonies than those without treatment (Figure 2f).

### Confirmation of the intracellular protein target of I-Trp in the cancer cell lines

To confirm that I-Trp can disrupt the CCT-*β *: *β*-tubulin complex in these cancer cells, we performed co-IP experiments using CCT-*β* antibody with the lysates of MDA-MB-231, MKN-45, HCT116, and Colo205 cells treated with I-Trp (5 or 10 *μ*M) for 6 h and then immunoblotted with *β*-tubulin antibody. With the I-Trp treatment, the trapped *β*-tubulin levels were lower than in untreated samples, indicating that I-Trp had disrupted the CCT-*β *: *β*-tubulin complex in these cancer cell lines (Figure 3a). With GADPH as a protein-loading control, we also confirmed that the input of *β*-tubulin and CCT-*β* in the experiments was equal with or without treatment. Although other tubulin-affecting anti-cancer drugs (e.g., paclitaxel) usually induce mitotic catastrophe, we have shown that I-Trp, unlike other tubulin-binding agents (e.g., paclitaxel), does not affect the polymerization/depolymerization of microtubules.^13^ Other than the polymerization assay, we examined the effects of I-Trp on the mRNA levels of CCT-*β* and *β*-tubulin. The RT-PCR experiments revealed that the mRNA levels of *β*-tubulin were not changed by I-Trp treatment (Supplementary Figures 1a–d). Also, according to the co-IP data (Figure 3a), the total amount of *β*-tubulin in the I-Trp-treated cells was not changed at the earlier time point (6 h), indicating disruption of CCT-*β *: *β*-tubulin complex occurred prior to apoptosis in these cancer cells. After 24–48 h of I-Trp treatment, cells underwent apoptosis to trigger proteasome activity for cellular protein degradation as reported previously.^14^

### I-Trp-induced ER stress in the cancer cells

In order to determine whether I-Trp-induced ER stress, we measured the proteasome activity in the target cell lines, as proteasome activation is associated with protein degradation resulting from cell apoptosis.^15, 16^ As shown in Figure 3b, the proteasome activities of MKN-45, HCT116, and Colo205 cells were elevated in an I-Trp dose-dependent manner, indicating I-Trp triggered a protein ubiquitination/degradation pathway; however, the effect was less apparent in MDA-MB-231 cells.

We then analyzed general ER stress biomarkers after I-Trp treatment in MDA-MB-231, MKN-45, HCT116, and Colo205 cells. In this study, we measured ER-related protein expression after I-Trp treatment for increasing periods of time (0, 6, 12, 24, 48, and 72 h). As shown in Figure 3c, BiP and PDI expression was increased after 24 h of I-Trp treatment in MKN-45 cells, but no differences were found in HCT116, Colo205, and MDA-MB-231 cells. Expression of calnexin was less affected by I-Trp in our experiments. The GADD153 transcriptional target, ER oxidase 1-like *α* (Ero1-L*α*), was also increased in a time-dependent manner after I-Trp treatment in MDA-MB-231, MKN-45, and Colo205 cells, but not in HCT116 cells.

Upregulation of ER stress transducer IRE1-*α* and PERK was evident in the western blot. In addition, we found that I-Trp promoted splicing of XBP1(U) into XBP1(S). PERK was also phosphorylated in these cancer cells after I-Trp treatment. The levels of CHOP increased after I-Trp treatment in MDA-MB-231, MKN-45, HCT116, and Colo205 cells. These are the hallmark proteins of ER stress,^17^ indicating that I-Trp-induced apoptosis mainly through ER stress. We also confirmed that media depletion did not affect these ER stress markers (Supplementary Figure 2).

### I-Trp treatment led to accumulation of intracellular Ca^2+^

Because the ER often releases Ca^2+^ during stress to amplify apoptotic signaling,^18^ we next analyzed the increase of intracellular Ca^2+^ upon I-Trp treatment. As shown in Figure 4a, I-Trp induced the intracellular Ca^2+^ mobilization in a dose-dependent manner in these cancer cell lines. Preloading BAPTA/AM, a powerful intracellular Ca^2+^ chelator, 1 h before I-Trp treatment reduced apoptosis (Figure 4b).

### Activation of apoptosis-associated proteins upon CCT-*β*: *β*-tubulin destruction

Caspase activation is thought to be critical for intracellular apoptotic signaling. In particular, the calcium signal activates caspase-3 and -9,^19^ while caspase-8 acts between death receptors and mitochondria.^20^ We thus investigated intracellular caspases activities in the I-Trp-treated cancer cells. The activities of caspases-2 to -9 were enhanced after incubation with I-Trp for 72 h (Figure 5a). Although caspase-8 activation is commonly induced by extracellular death signals, recent study shows that ER stress can also activate caspase-8 to induce cell apoptosis.^21^ As shown in Figure 5b, I-Trp treatment increased cleaved caspase-3, -7, -9, and PARP, an intracellular downstream target of caspase-3/-7, in the target cells. These cleaved forms indicate that intracellular caspase cascade is involved in mediating apoptotic signaling induced by I-Trp interrupting constitutive *β*-tubulin/CCT-*β* complexes. We also examined the caspase activation at earlier (<72 h) time periods (0, 12, 24, and 48 h) with I-Trp treatment at EC_50_ concentrations. As shown in Figure 5c, I-Trp treatment triggered the time-dependent activation of intracellular caspases-3 at 24–48 h, especially 48 h.

### I-Trp upregulated the expression of pro-apoptotic proteins and inhibited the expression of anti-apoptotic proteins

The balance between different ER-localized Bcl-2 family proteins regulates the activation of ER signaling pathways and thus cell survival. ER-localized anti-apoptotic proteins, such as Bcl-2 and Bcl-xL, suppress a variety of apoptosis-inducing stimuli, including ER-localized pro-apoptotic proteins Bax/Bak and various BH3-only proteins.^22, 23, 24^ In measuring the expression levels of pro-apoptotic and anti-apoptotic proteins, especially Bcl-2 family proteins, we found that I-Trp increased the expression of pro-apoptotic proteins such as Bax, Bad, and Bak. In addition, I-Trp decreased the expression of anti-apoptotic proteins such as Bcl-2 and Bcl-xL (Figure 5d).

### I-Trp-induced apoptosis through the activation of MAPKs

MAPKs, including ERK1/2, p38, and JNK, play essential roles in the regulation of cellular response, including cell survival, apoptosis, proliferation, and differentiation.^25, 26, 27^ MAPKs activation has been implicated in the regulation of ER stress-induced cell death.^24, 28, 29, 30^

I-Trp-induced cell death may proceed via the kinase function of IRE1-*α*, involving the activation of MAPK family proteins, such as p38 and JNK. Activated JNK can promote the activation of Bim and inhibition of Bcl-2, whereas p38 can activate CHOP.^31^ Both p38 and JNK can promote the activation of pro-apoptotic protein Bax.^32^ Therefore, we investigated whether MAPKs are involved in the cell apoptosis caused by I-Trp. We pretreated the cancer cells with I-Trp for different time periods and measured phosphorylated ERK, JNK, and p38 using western blot. We found marked ERK phosphorylation in MDA-MB-231 and HCT116 cells. On the other hand, we did not observe ERK phosphorylation in MKN-45 cells. JNK was phosphorylated in MKN-45 cells but not in MDA-MB-231 and HCT116 cells. In Colo205 cells, the phosphorylation levels of ERK and JNK were slightly changed. In addition, I-Trp increased p38 phosphorylation in all target cancer cell lines (Figure 6a).

Next, we used ERK1/2 inhibitor U0126,^33, 34^ JNK inhibitor SP600125,^35, 36^ and p38 inhibitor SB203580^37^ to confirm the involvement of MAPKs in I-Trp-induced apoptosis. We pretreated the cancer cell lines with 0.5–10 *μ*M U0126, 1–10 *μ*M SP600125, or 1–10 *μ*M SB203580 for 1 h and subsequently treated them with I-Trp to detect the sub-G_0_ cell accumulation by flow cytometry. As shown in Figure 6b, we found that U0126 could not reverse the I-Trp-induced apoptosis in MKN-45 cells, thereby confirming the lack of ERK phosphorylation activation. SP600125 could not reverse I-Trp-induced apoptosis in MDA-MB-231 and HCT116 cells, either (Figure 6c). In contrast, the p38 inhibitor SB203580 (Figure 6d) reduced the percentage of I-Trp-induced apoptotic cells. Our results show that induction of cell apoptosis by I-Trp requires activation of the MAPK family.

### Synthesis and evaluation of I-Trp analogs

**Figure 1:**
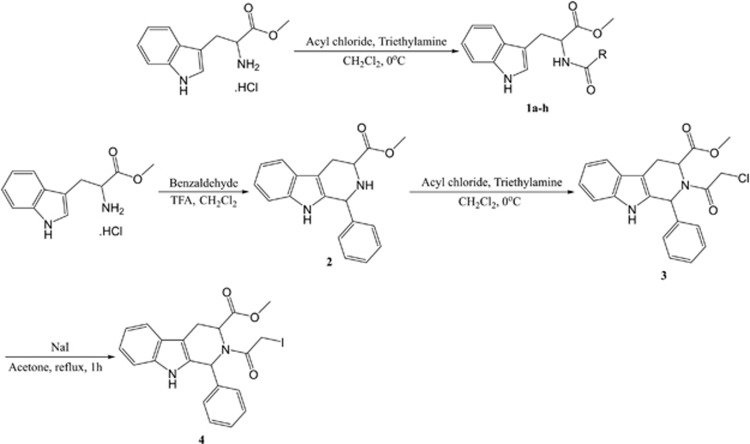
Synthesis of I-Trp and its analogs. The reaction conditions and the reagents used are shown in the Figure

**Figure 2:**
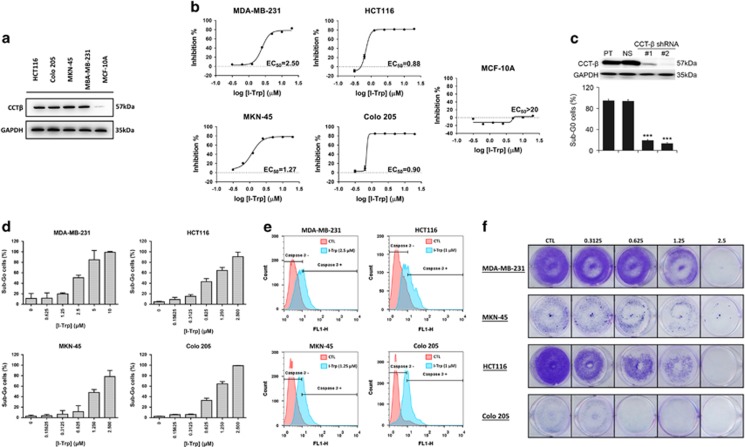
Effect of I-Trp on CCT-*β* overexpressed cancer cells. (**a**) The higher expression levels of CCT-*β* in the selected cancer cells, including MDA-MB-231, MKN-45, Colo205, and HCT116, assessed by immunoblotting with CCT-*β* antibody. (**b**) EC_50_ of these cancer cells measured using MTT assays. Apoptosis was measured 72 h after treatment. (**c**) Western blot analysis for CCT-*β* protein and the loading control GAPDH derived from MDA-MB-231 cells transfected without (parental, PT) or with non-silencing (NS) or two independent CCT-*β* shRNA clones (#1 and #2). Sub-G0 cell populations were determined by PI-based flow cytometric analysis on the cells treated with I-Trp at 10 *μ*M for 72 h. Data from three independent experiments are presented as mean±S.D. The symbol *** denotes *P*<0.001. (**d**) I-Trp-induced concentration-dependent Sub-G_0_ accumulation of these cancer cells. After treatment with different concentrations of I-Trp for 72 h, cell apoptosis was assessed via PI-based flow cytometric analysis. (**e**) I-Trp-induced cleaved caspase-3 accumulation after treatment with different EC_50_ concentrations of I-Trp for 72 h on the four cancer cell lines. Cell apoptosis was assessed via an active caspase-3 staining-based flow cytometric analysis. (**f**) Long-term colony formation assays for these cancer cells treated with 0.31 to 2.5 *μ*M of I-Trp for 7 days

**Figure 3:**
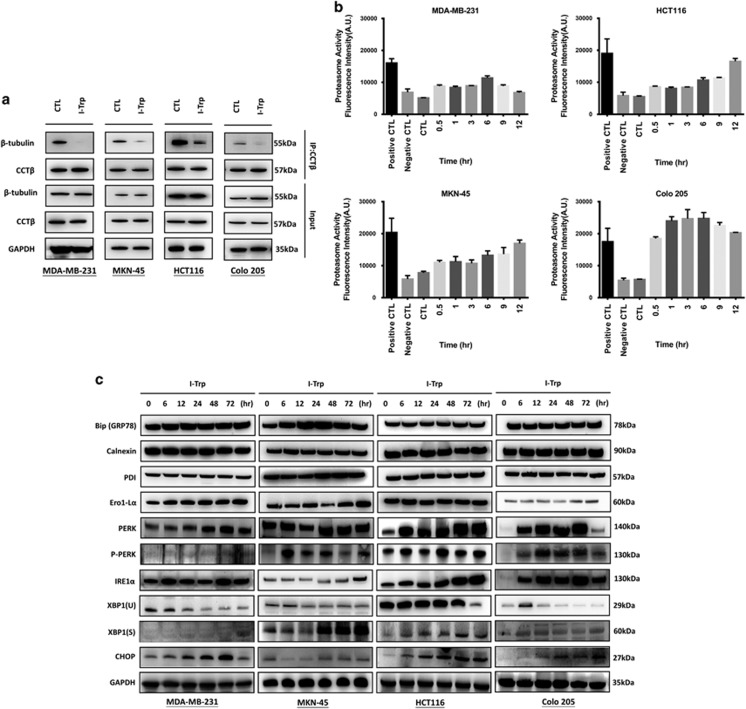
Disruption of CCT-*β *: *β*-tubulin by I-Trp. (**a**) Using CCT-*β* antibody to co-IP the complex, and then detecting the quantity of trapped *β*-tubulin with western blotting using *β*-tubulin antibody. The trapped *β*-tubulin in the cancer cells was reduced in quantity upon I-Trp treatment, indicating I-Trp blocked the PPI. (**b**) Targeting the CCT-*β* : *β*-tubulin complex with I-Trp promotes proteasome activity. The cancer cells were treated with I-Trp for the designated time periods. Cell lysates were examined using the proteasome activity assay. (**c**) The changed expression levels of ER stress protein markers upon I-Trp treatment

**Figure 4:**
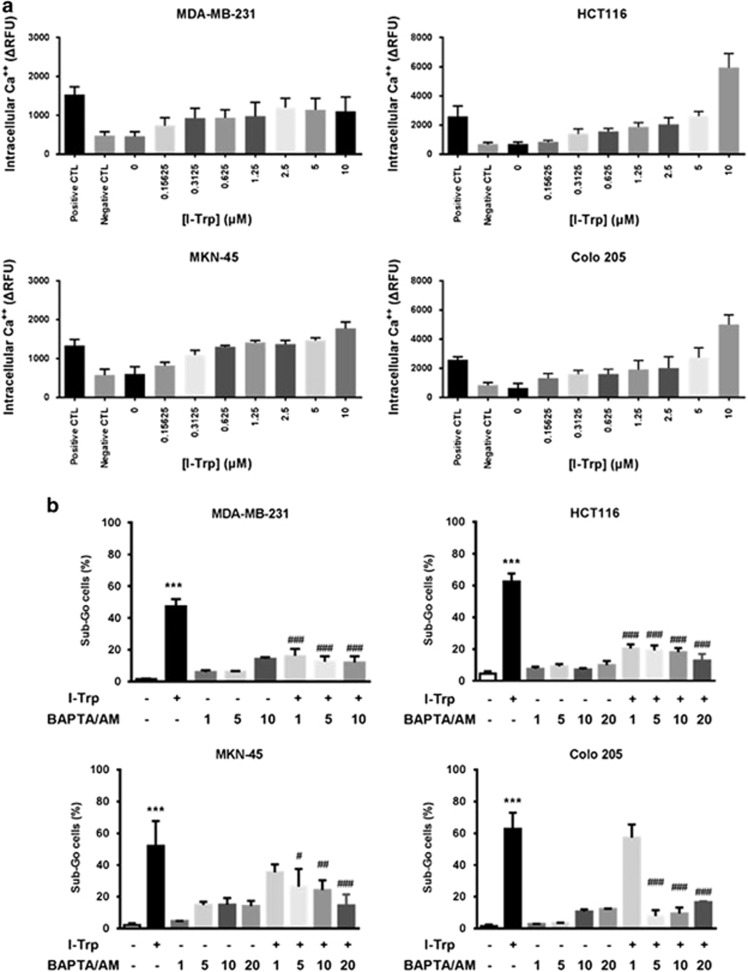
Effect of I-Trp on the levels of intracellular Ca^2+^. (**a**) I-Trp induces intracellular Ca^2+^ release in a concentration-dependent manner. Cells were pretreated with BAPTA/AM (negative control) for 1 h, with MCN-A-343 (positive control) or different concentrations of I-Trp and incubated with Fluo-4 for 40 min. After incubation, the fluorescence signal was immediately measured using a fluorescence microplate reader by excitation at 509 nm and emission at 516 nm. (**b**) Cells were pre-incubated with BAPTA/AM (1–20 *μ*M) for 1 h prior to treatment with EC_50_ concentrations of I-Trp for 72 h. Cell apoptosis was assessed via PI-based flow cytometric analysis. Data are expressed as mean±S.D. ***P<0.001, significant difference as compared to control. ^#^*P*<0.05, ^##^*P*<0.01 and ^###^*P*<0.001 significant difference as compared to I-Trp alone

**Figure 5:**
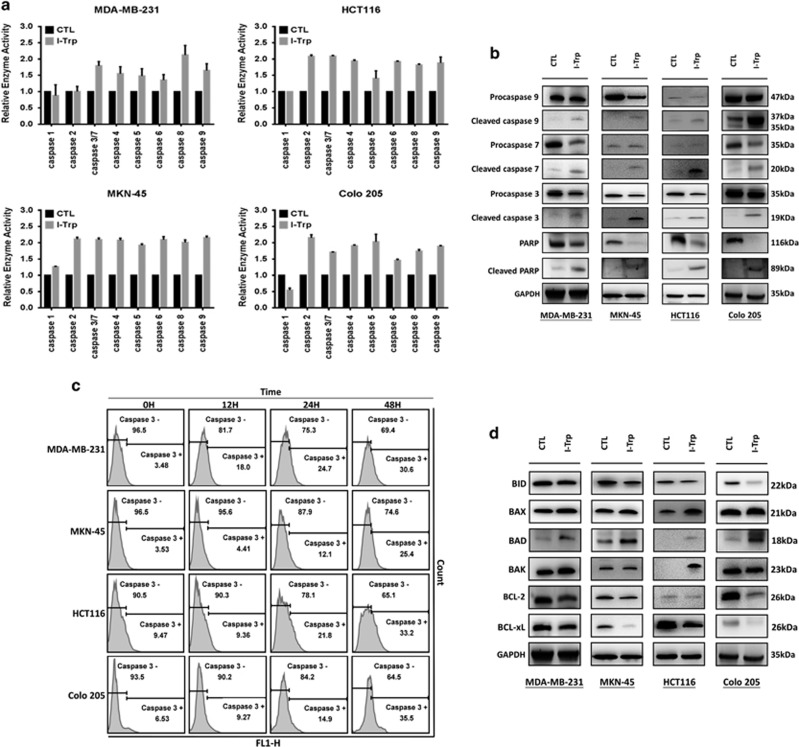
Caspase activation induced by I-Trp in the cancer cells. (**a**) The cells were treated with the EC_50_ concentrations of I-Trp. Levels of caspase-1, -2, -3/7, -4, -5, -6, -8, and -9 were assessed 72 h after I-Trp treatment. (**b**) Quantification of cellular procaspase-9, caspase-9, procaspase-7, caspase-7, procaspase-3, caspase-3, PARP, and cleaved PARP upon I-Trp treatment by western blot analysis using their antibodies. (**c**) The cleaved caspase-3 was accumulated after treatment with different EC_50_ concentrations of I-Trp for 0–48 h. Cell apoptosis was assessed via an active caspase-3 staining-based flow cytometric analysis. (**d**) Quantification of cellular anti-apoptotic and pro-apoptotic proteins upon I-Trp treatment by western blot analysis

**Figure 6:**
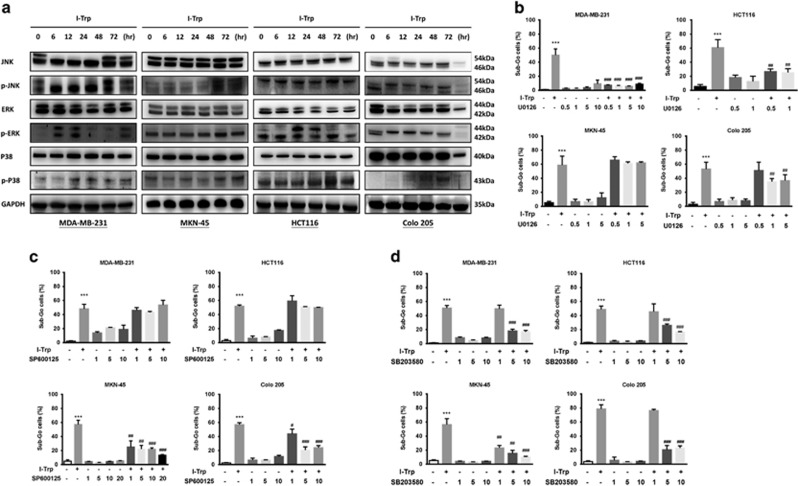
I-Trp promotes ER stress-induced cell apoptosis via the MAPK pathway upon CCT-*β *: *β*-tubulin complex destruction. (**a**) Quantification of cellular JNK, p-JNK, ERK, pERK, p38, and p-p38 upon I-Trp treatment by western blot. Using the kinase inhibitors to test the pathway of cell apoptosis induced by I-Trp. Commercially available ERK inhibitor U0126 (**b**), JNK inhibitor SP600125 (**c**), and P38 inhibitor SB203580 (**d**) were used to inhibit the kinases, respectively, and the sub-G_0_ cells were accessed using flow cytometer. Data are expressed as mean±S.D. ***P<0.001, significant difference as compared to control. ^#^*P*<0.05, ^##^*P*<0.01, and ^###^*P*<0.001 significant difference as compared to I-Trp alone

As the iodo-moiety is not desirable in drug discovery due to its reactivity, we explored other possible reactive electrophiles. The synthesis of these I-Trp analogs are shown in Figure 1 and their EC_50_ against the cell lines are summarized in Table 1. We initially synthesized compounds **1a**–**h**. Among them, only compound **1b** with bromo-substituent at R was almost equipotent as I-Trp (**1a**) in killing these cancer cells. Compounds with a poor leaving group at R were inactive.

We hypothesized that the binding energy of the molecule could be lost due to the flexibility of the alkyl side chain of tryptophan. Cyclizing it to a tetrahydroisoquinoline derivative could regain the lost entropy and thus promote inhibition. Based on this hypothesis, we converted compound **1c**, which was mostly inactive, to tetrahydroisoquinoline derivative **3**, which effectively killed cancer cells. Substituting CH_2_Cl with CH_2_I further increased the potency of compound **4**. Though **4** was slightly less (~twofold) potent than doxorubicin, it was significantly less (~fivefold) toxic compared to doxorubicin as demonstrated on MCF-10A.

## Discussion

In this study, we demonstrated that a TNBC MDA-MB-231, colorectal cancers Colo205 and HCT116, and a gastric cancer MKN-45 express higher levels of CCT-*β* than normal cells and many other cancer cell lines. I-Trp, which is known to disrupt the intracellular CCT-*β *: *β*-tubulin complex,^14^ kills these cancers with selectivity, relative to the normal cell MCF-10A. Knockdown of CCT-*β* reduced the I-Trp cytotoxicity. The EC_50_ of I-Trp against MDA-MB-231, Colo205, HCT116, and MKN-45 were measured by MTT assays to be 2.50, 0.90, 0.88, and 1.27 *μ*M, respectively, and flow cytometric assays show the sub-G_0_ accumulation of the treated cancer cells in a dose-dependent manner, consistent with the MTT assays. These data together demonstrate the feasibility of using a PPI inhibitor to treat CCT-*β* overexpressed cancers through disrupting the CCT-*β *: *β*-tubulin complex.

ER is a specialized organelle involved in crucial cellular functions, including protein folding and Ca^2+^ storage/signaling. Alterations in the ER folding environment cause the accumulation of misfolded proteins in the ER lumen, leading to ER stress.^38, 39^ To restore ER homeostasis, cells activate the unfolded protein response, a signaling pathway governed by three major ER stress sensors, PERK, IRE1, and activating transcription factor 6. Together, these signaling pathways coordinate a temporal shutdown in protein translation and induce a complex program of gene transcription to restore the folding capacity of the ER. However, chronic or too severe ER stress will engage apoptosis.^39, 40^ We show that the apoptosis induced by I-Trp in these cancer cells is probably through ER stress and caspase activation. The proteasome activity was increased alongside intracellular Ca^2+^ release from the ER. The disruption of the CCT-*β *: *β*-tubulin complex likely impedes the formation of microtubules, then affecting morphology, trafficking, and expansion of ER,^41, 42^ thereby promoting the accumulation of unfolded proteins in the ER and the initiation of ER stress response. Alternative mechanisms cannot be excluded because CCT-*β* regulates many other proteins beside *β*-tubulin. Furthermore, all the caspases except caspase-1 are activated by I-Trp treatment in these cancer cells. With PARP cleavage, these hallmarks of apoptosis indicate that I-Trp induces a caspase-dependent mechanism through ER stress.

ER-localized anti-apoptotic proteins, such as Bcl-2 and Bcl-xL, suppress a variety of apoptosis-inducing stimuli, including ER-localized pro-apoptotic proteins Bax/Bak and various BH3-only proteins. Both ER and mitochondrial apoptosis signaling pathways lead to Bax and Bak activation and apoptosis, although there are differences between the two apoptosis signaling pathways.^22, 43^ Therefore, pro-apoptotic proteins, such as Bax, Bad, and Bak, were increased, whereas the anti-apoptotic proteins, such as Bcl-2 and Bcl-xL, were decreased on I-Trp treatment in these CCT-*β* overexpressed cancer cells.

**Figure 7:**
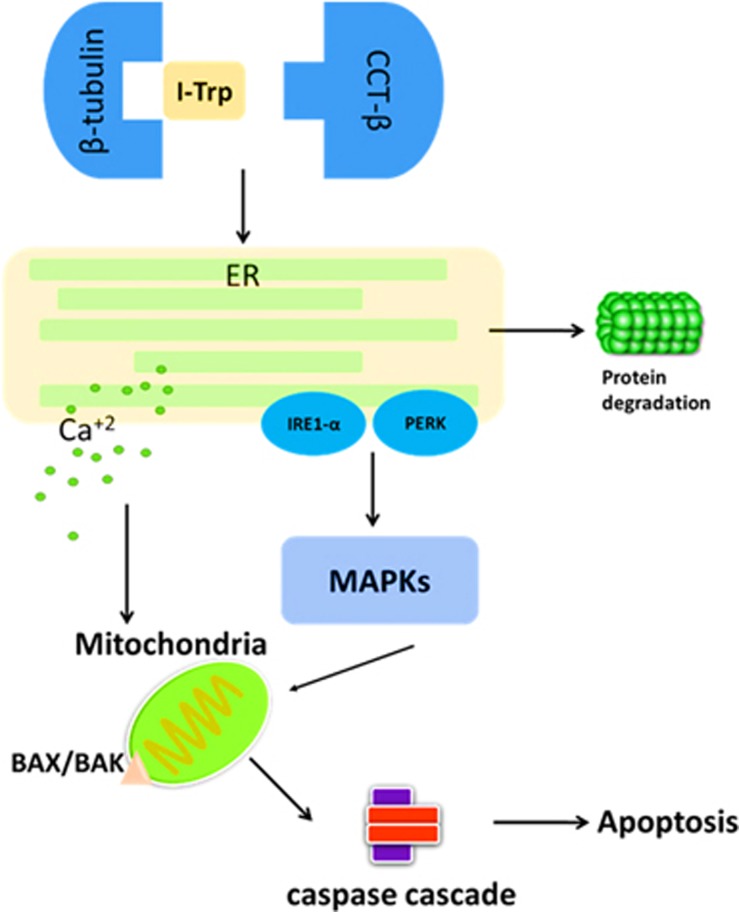
The apoptotic molecular signaling pathway. Both protein ubiquitination/degradation and the ERAD pathways are activated through the MAPKs, at the onset of CCT-*β *: *β*-tubulin complex disruption

Previous studies have shown that under different cellular stresses, Bax and Bak can directly bind with IRE1-*α* on the cytoplasmic side of the ER membrane. IRE1-*α* then recruits ASK1, leading to activation of the pro-apoptotic signaling kinase JNK and downstream pro-apoptotic transcription factors c-Jun and p38 MAPK. Activated JNK can promote activation of Bim and inhibition of Bcl-2, whereas p38 can activate CHOP.^31, 44^ Both p38 and JNK can promote activation of pro-apoptotic protein Bax.^32^ In this study, we further demonstrate MAPKs involvement in the apoptosis induced by I-Trp. In summary, our mechanistic studies show that I-Trp leads the cancer cells toward ER stress with elevated intracellular Ca^2+^ levels, activation of MAPKs, and then mitochondrial destruction, ultimately activating caspases toward apoptosis (Figure 7). Our study therefore unveils the signaling pathways induced by disrupting the PPI.

Overall, treatment of TNBC, gastric cancer, or colorectal cancer is an ongoing clinical challenge, and how to improve survival remains an important component of developing new agents. TNBC is the major clinical challenge in breast cancer therapy. Cytotoxic chemotherapy is still the mainstay of treatment for patients with TNBC. Although some patients respond, the treatment is toxic, and a high percentage of patients treated in the early stage eventually relapse. In the metastatic setting, the survival of patients remains dismal. Currently, PARP inhibitors, mTOR inhibitors, PI3K inhibitors, Src inhibitors, growth factor inhibitors, and AR antagonists are under evaluation in clinical trials for TNBC treatment. Also lethal, gastric cancer is the third leading cause of cancer death worldwide. Surgery is the primary strategy if gastric cancer is diagnosed at an early stage, whereas chemotherapy and radiotherapy tend to be used at a later stage. In recent years, two monoclonal antibodies (trastuzumab and ramucirumab) and a small molecule (apatinib approved by CFDA) have improved survival in patients with gastric cancer. Other small-molecule agents, such as AZD4547 (FGFR inhibitor) and BYL719 (PI3K*α* inhibitor), are now in phase II clinical trials. In colorectal cancer, surgery is usually the primary treatment, with some applications for adjuvant chemotherapy. Targeted therapies are currently in use to treat colorectal cancer with the monoclonal antibodies bevacizumab, cetuximab, and panitumumab; however, they are only effective in patients with tumors lacking KRAS mutations. Regorafenib, an oral drug that targets multiple tyrosine kinases, was approved in 2012 as second-line therapy, but it provides limited survival benefit by increasing the survival from 5 months with placebo to 6.4 months. Here we present a novel strategy by blocking the CCT-*β *: *β*-tubulin PPI to selectively kill these CCT-*β* overexpressed malignant cancers. We also establish the structure–activity relationship of I-Trp in killing these cancer cell lines. These results hold the promise for the development of more potent inhibitors. We are proceeding to identify reversible inhibitors for the PPI in an effort to increase the efficacy of cancer therapy in TNBC, gastric cancer, and colorectal cancer.

## Materials and methods

### Materials

All chemicals used for organic synthesis and protein A-agarose were purchased from Sigma-Aldrich (St. Louis, MO, USA). The antibody for CCT-*β* was purchased from Abcam Inc. (Cambridge, MA, USA). The antibody for *β*-tubulin was purchased from Thermo Fisher Scientific Co. (Fair Lawn, NJ, USA). Other antibodies were purchased from Cell Signaling (Danvers, MA, USA). BAPTA/AM (1,2-*bis*(o-aminophenoxy) ethane-N,N,N′,N′-tetraacetic acid) and U0126 were purchased from Calbiochem (San Diego, CA, USA). Other drugs were purchased from the following: SB203580 (Selleckchem, Houston, TX, USA) and SP600125 (Selleckchem). Caspase and proteasome activity assay kits were purchased from BioVision (Milpitas, CA, USA).

### Syntheses of I-Trp (1a) and its analogs

I-Trp (**1a**) and its analogs (**1b**–**h**, **3**, and **4**) were synthesized according to Figure 1. For the synthesis of compounds **1a**–**h**, the suspended solution of D-tryptophan methylester hydrochloride (1.0 eq) in 3 ml anhydrous dichloromethane was cooled to 0 °C, and then triethylamine (3.0 eq) was added and stirred for 5 min. Substituted acylchloride was gradually added to this cold mixture and the stirring was continued for 3 h, maintaining the temperature at 0 °C. After confirming completion of the reaction by TLC, the reaction mixture was poured into a separating funnel and washed with water and then brine. The dichloromethane layer was then collected, and then dried over MgSO_4_ to obtain crude compound, which was later purified chromatographically. For synthesis of compounds **3** and **4**, the suspended solution of D-tryptophan methylester hydrochloride (1.0 eq) in 3 ml anhydrous dichloromethane was cooled to 0 °C, then benzaldehyde (1.0 eq) was added and stirred for 5 min. An aliquot of 0.2 ml of TFA was added to this cold mixture, and the reaction was allowed to climb to room temperature gradually. The reaction mixture was stirred for 24 h, then transferred to a separating funnel and washed with water followed by sat. NaHCO_3_ solution. The dichloromethane layer was dried over MgSO_4_ to obtain crude compound as *cis* and *trans* isomers, which were later purified chromatographically to obtain **2**. Compound **2** was then treated with chloroacetyl chloride as described earlier (synthesis of **1a**–**h**) to obtain **3**. Compound **3** was dissolved in acetone, then sodium iodide (3.0 eq) was added and refluxed for 1 h. Acetone was removed under vacuum and the crude compound **4** was purified using column chromatography.

### Cell culture

MDA-MB-231 and MKN-45 cell lines were obtained from the Bioresource Collection and Research Center in Taiwan and Japanese Collection of Research Bioresources Cell Bank, respectively. HCT116 and Colo205 cell lines were obtained as a gift from Dr. Chiung-Tong Chen, National Health Research Institute, Taiwan. The cells were cultivated in DMEM, RPMI1640, and McCoy’s 5A supplemented with 10% fetal bovine serum (FBS) and 250 units/ml of penicillin/streptomycin solution. All cell culture media and supplements were purchased from Gibco (Carlsbad, CA, USA). The cells were maintained in the presence of 5% CO_2_ at 37 °C.

### Western blotting

The cell lysates (100 μg) were boiled for 5 min in SDS sample buffer (62.5 mM Tris-HCl pH 6.7, 1.25% SDS, 12.5% glycerol, and 2.5% *β*-mercaptoethanol), and the proteins were separated via SDS-PAGE. For western blotting, proteins were transferred onto a PVDF membrane (Millipore, MA, USA) and incubated with different antibody agents. After incubation with horseradish peroxidase (HRP)-conjugated secondary antibody, immuno-reactive protein bands were visualized using the enhanced chemiluminescence (ECL) system (Amersham Bioscience, Tokyo, Japan).

### Apoptosis assessment

Cells (3 × 10^5^) were fixed in 70% ice-cold EtOH for 30 min at RT. Cells were washed with PBS once and then incubated with propidium iodide (PI) staining solution (PBS containing 0.1 % BSA, 0.1% RNase A, and 20 ng/ml PI) for 30 min at RT in the dark. After the incubation, the cells were analyzed by flow cytometry to assess their DNA content.

### Caspase-3 apoptosis assay

After incubation with different EC_50_ concentrations of I-Trp for 12, 24, 48, and 72 h, cells were collected by trypsinization fixed with formaldehyde for 10 min at 37 °C (2 × 10^6^ cells/ml), permeabilized with methanol, and finally resuspended in incubation-buffer (FBS: PBS 1: 200). The FITC-conjugated monoclonal antibody (#9661; Cell Signaling Technology) that detects the endogenous large fragment (17/19 kDa) of the activated caspase-3 was used. Cells were analyzed by flow cytometry (BD FACS Calibur Flow Cytometer) with FACS Diva software. Untreated cells were used as a negative control.

### Determination of proteasome activity

After being treated with I-Trp for designated time periods, cells were collected and resuspended in cell lysis buffer (BioVision). Aliquots of cell lysates (100 *μ*g) were incubated with 1 *μ*M of fluorescent proteasome substrate (BioVision) for 30 min at 37 °C. The fluorescent intensity was read using a fluorimeter with an exciting wavelength at 355 nm and emission wavelength at 405 mm and presented as relative proteasome activity.

### Measurement of intracellular Ca^2+^ signaling

We used a Fluo-4 direct calcium assay kit from Invitrogen (catalog item F10471) to measure the intracellular calcium response to I-Trp in target cancer cell lines. Cancer cell lines were cultured in 96-well plates with media containing 10% FBS. After reaching confluence, cancer cells were treated with MCN-A-343 (positive control), BATPA (negative control), or I-Trp and incubated with Fluo-4 direct calcium reagent loading solution for 40 min. After incubation, the fluorescence signal was immediately measured using a fluorescence microplate reader at excitation and emission of 509 and 516 nm, respectively.

### Determination of caspases activities

The caspase activity assay was performed according to the manufacturer’s guidelines (BioVision). Briefly, cell lysates (200 *μ*g) were diluted in 50 *μ*l of cell lysis buffer (supplied by the kit). Equal volumes of the 2 × reaction buffer (supplied by the kit) containing 10 mM DTT were added to the cell lysates. Subsequently, 50 *μ*M of fluorescent dye-conjugated caspase substrate was individually added to the designated caspase activity assay. After a 2-h incubation, free fluorescent dyes in the solution were read in a fluorimeter equipped with a 385-nm excitation filter and 510-nm emission filter. Fold increases in caspase activity were determined by comparing these results with the level of the untreated control.

## Figures and Tables

**Table 1:** EC_50_ of the synthesized compounds against cancer and normal cells

*Compound*	*R*	*EC*_*50*_ *(μM)*				
		*MDA-MB-231*	*MKN-45*	*HCT116*	*Colo205*	*MCF-10A*
**1a** (I-Trp)	–CH_2_I	2.50	1.27	0.88	0.90	>20
**1b**	–CH_2_Br	3.75	2.5	1.1	1.6	>20
**1c**	–CH_2_Cl	19.5	>20	>20	>20	>20
**1d**	–CHCl_2_	>20	>20	>20	>20	>20
**1e**	–CH_2_NO_2_	>20	14.1	20.9	15.9	>20
**1f**	–CH_2_CF_3_	>20	>20	>20	>20	>20
**1g**	–CF_3_	>20	>20	>20	>20	>20
**1h**	–CH_2_CN	>20	>20	>20	>20	>20
**3**	–CH_2_Cl	2.7	4.7	3.3	8.1	>20
**4**	–CH_2_I	0.59	1.6	0.54	0.99	~20
Doxorubicin		0.46	0.24	0.26	0.50	3.8
