# Supplementary material for: Disrupting CCT-β : β-tubulin selectively kills CCT-β overexpressed cancer cells through MAPKs activation
Source: Cell Death Dis. 2017 Sep 14;8(9):e3052–. doi: 10.1038/cddis.2017.425 (PMC5636972; doi:10.1038/cddis.2017.425)
Supplement: Supplementary Figures [file cddis2017425x1.docx]

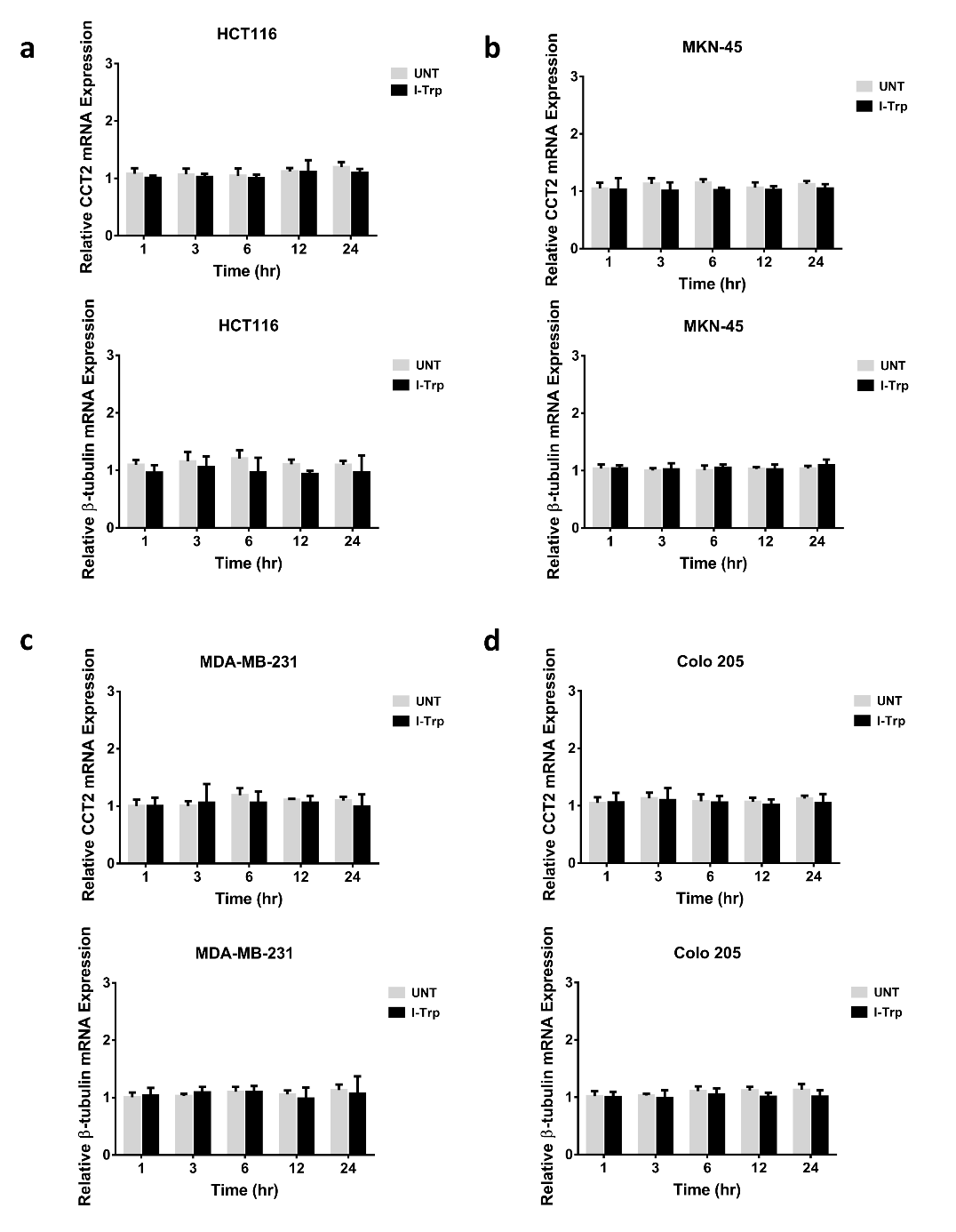

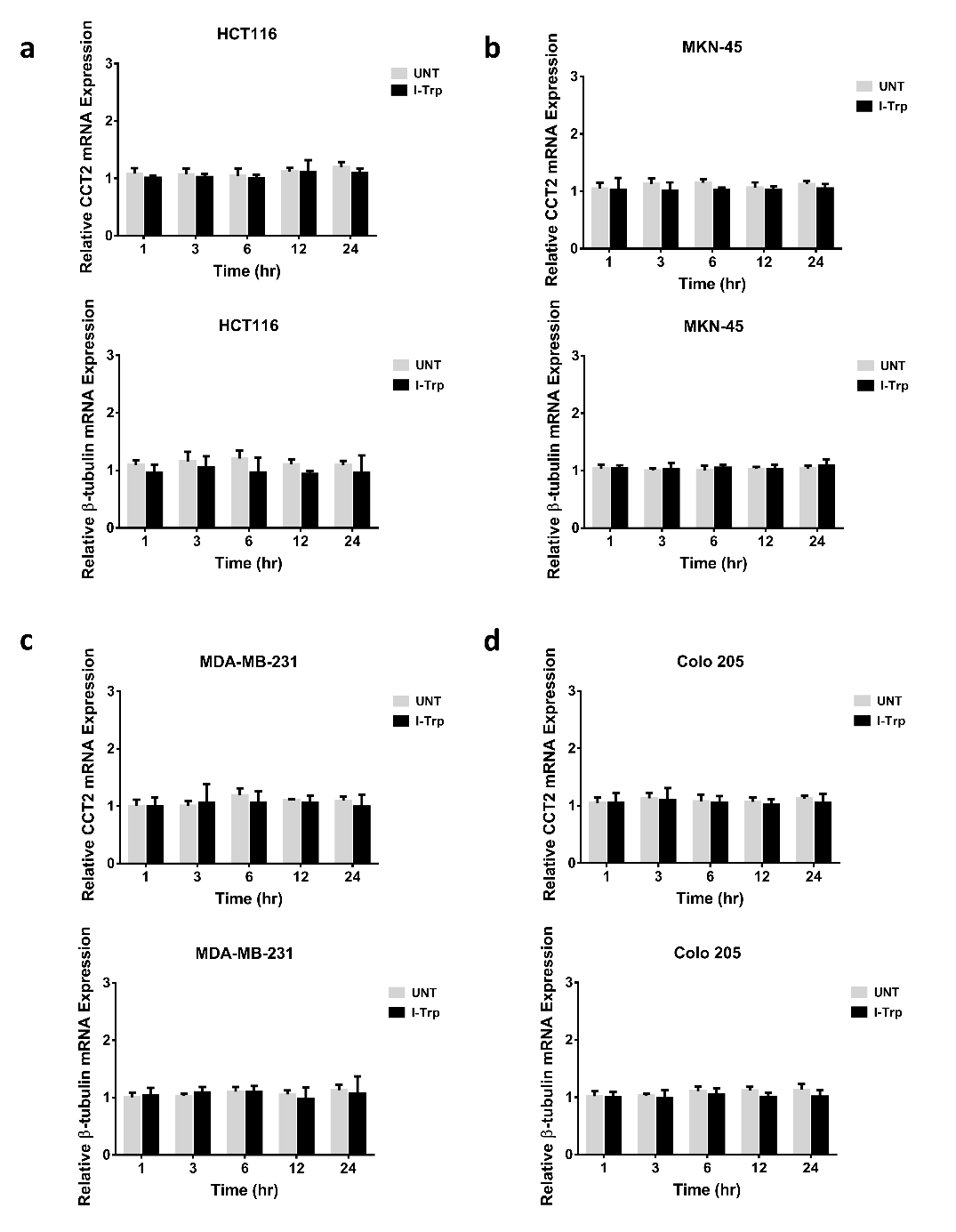


Supplementary Figure 1: I-Trp treatment did not change the mRNA levels of CCT-β and β-tubulin. Real time qPCR analysis for CCT-β and β-tubulin was performed in the cancer cells, HCT116 (**a**), MKN-45 (**b**), MDA-MB-231 (**c**), and Colo205 (**d**). Mean values ± S.D. from three independent samples are shown. The RT-PCR experiments reveal that the mRNA levels of CCT-β and β-tubulin were not changed after I-Trp treatment in the tested cancer cells.


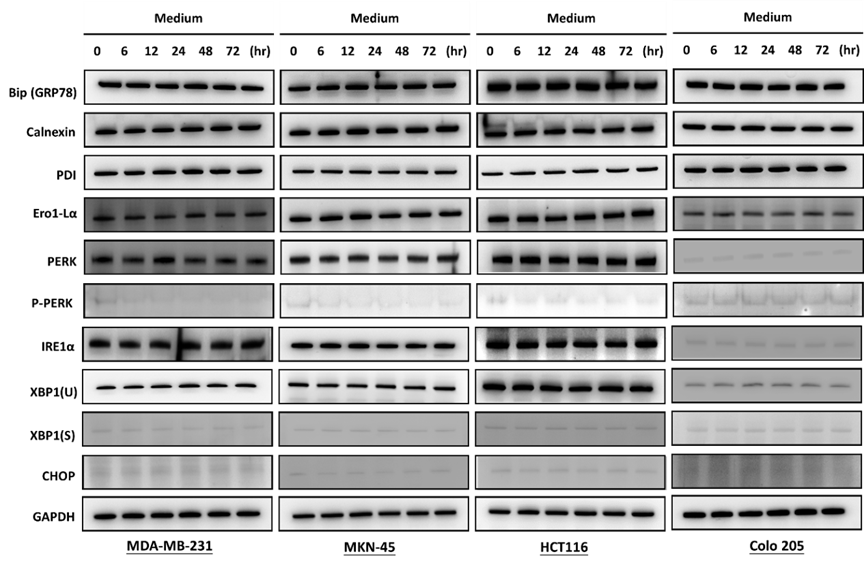


Supplementary Figure 2: The medium depletion did not affect the expression of ER stress markers. The cancer cells were collected after 0–72 h in the absence of I-Trp and the expression of ER stress markers was analyzed using Western blot. GAPDH was used as a loading control. Some ER stress proteins were present from the beginning and the media depletion did not affect the expression of ER stress markers.
